# Inhibitory Receptor Diffusion Dynamics

**DOI:** 10.3389/fnmol.2019.00313

**Published:** 2019-12-19

**Authors:** Stephanie A. Maynard, Antoine Triller

**Affiliations:** Institute of Biology of Ecole Normale Supérieure (IBENS), PSL Research University, CNRS, Inserm, Paris, France

**Keywords:** diffusion-trapping, inhibitory synapse, GABA_A_ receptor, glycine receptor, gephyrin, single-particle tracking

## Abstract

The dynamic modulation of receptor diffusion-trapping at inhibitory synapses is crucial to synaptic transmission, stability, and plasticity. In this review article, we will outline the progression of understanding of receptor diffusion dynamics at the plasma membrane. We will discuss how regulation of reversible trapping of receptor-scaffold interactions in combination with theoretical modeling approaches can be used to quantify these chemical interactions at the postsynapse of living cells.

## Introduction

Synaptic organization is a dynamic multiscale process in neuronal cell networks. The role of receptor diffusion-trapping in the plasma membrane is now understood to be a molecular mechanism resulting from chemical interactions and is crucial for synapse formation, stability, and plasticity in neurons.

The fluid mosaic model postulated by Singer and Nicolson (1972) reflects the thermodynamics and interactions of the individual components which have a critical role in the composition and organization of biological membranes. This idea was further enforced when, in 1974, the receptor protein rhodopsin was shown to be in constant motion in the lipid bilayer (Poo and Cone, 1974). Beside molecular turnover, it became obvious that synaptic molecular components were dynamic, enabling activity-dependent regulation of synaptic functions. The importance of molecular turnover at the synapse, as a regulator of synaptic strength and memory, was suggested by Crick (1984). Crick noted that the individual molecular components of the synapse were subject to characteristic times of turnover shorter than that of memory. Crick postulated that post-translational modifications of the molecules at the synapse could explain a longer-term persistent state of synaptic strength contributing to memory, despite a molecular turnover within days. Since then, there has been a concentrated effort into uncoupling membrane composition, diffusion dynamics and activity-dependent synaptic regulation with long-term structural stability.

This review will focus on the recent advances in our understanding of molecule dynamics in inhibitory synapses, covering technological advancements that have enabled probing of receptor and scaffold protein dynamics, organization and regulation.

## Receptor Diffusion-Trapping Dynamics in the Inhibitory Synapse

### Membrane Receptor Insertion

Underlying fundamental processes controlling synaptic receptor delivery and removal, and the implications of these in synaptic strength have been of intense interest over the last 20 years. It was previously known that regulation of receptor number at the post synapse influenced plasticity at both excitatory and inhibitory synapses (e.g., Nusser et al., 1998; Hayashi et al., 2000; reviewed in Turrigiano, 2000). It was originally postulated that the dynamic turnover was driven exclusively by endocytosis and exocytosis of receptors and scaffold molecules to the membrane following *de novo* receptor synthesis or recycling. GABA_A_ receptor (GABA_A_R) exocytosis and endocytosis *via* a clathrin-mediated pathway demonstrated exchange between the surface and intracellular compartments of the synapse (Kittler et al., 2000). Further, it was shown glycine receptor (GlyR) exocytosis occurred predominantly at extrasynaptic sites in the cell body and initial portion of dendrites in spinal cord neurons, and that this exocytosis was not directed or synapse-specific (Rosenberg et al., 2001). GABA_A_R exocytosis was also shown to be extrasynaptic followed by recruitment to synapses *via* lateral diffusion in the membrane in hippocampal neurons (Thomas et al., 2005; Bogdanov et al., 2006). Studies of excitatory synapses have showed AMPAR GluR1 subunits are initially inserted at extrasynaptic sites, whereas the GluR2 subunit is inserted in spines closer to synapses (Passafaro et al., 2001) and thus subunit specificity may further regulate receptor delivery. Further, in hippocampal pyramidal neurons, AMPARs were shown to enter spines preferentially following membrane insertion in the adjoining dendritic shaft (Yudowski et al., 2007). The balance of exocytosis and endocytosis regulates the number of postsynaptic receptors and has long been regarded as the main cellular mechanism underlying long-term potentiation (LTP) and long-term depression (LTD) (Mammen et al., 1997; Nishimune et al., 1998; Lüthi et al., 1999; Song and Huganir, 2002; Park et al., 2004; Tanaka and Hirano, 2012; Fujii et al., 2018).

### Membrane Receptor Diffusion

**Figure 1:**
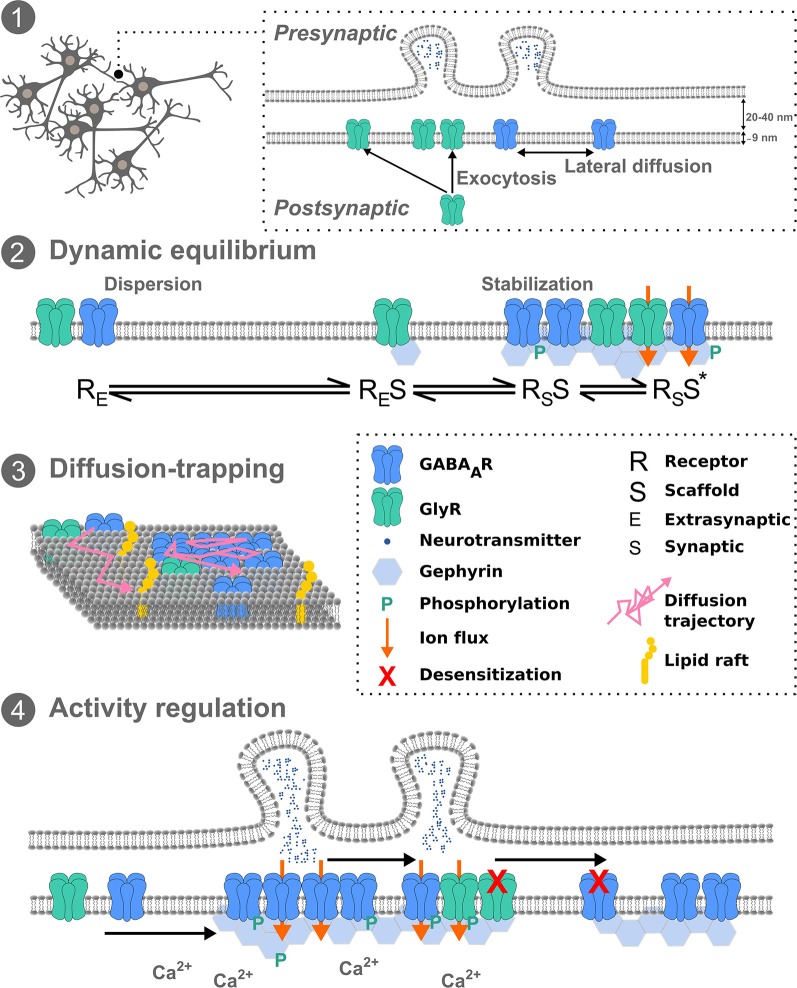
Inhibitory receptor diffusion-trapping. (1) Overview schematic of pre- and postsynaptic inhibitory neuronal membranes, exocytosis, and lateral diffusion. (2) The dynamic equilibrium between stabilized and freely mobile receptors, at the synapse and extrasynaptically, respectively. (3) Receptor diffusion-trapping depends not only on chemical interactions with synaptic components but also on non-specific obstacles, such as lipid rafts, leading to molecular crowding. (4) Activity regulation of receptor mobility can affect post-translational modifications of receptors and scaffold proteins and subsequently their immobilization at synapses. Exchange of receptors between synapses can fine-tune network activity.

However, in addition to receptor exocytosis and endocytosis, lateral receptor diffusion and trapping within the postsynaptic membrane has since been established as a key mediator of synaptic strength and plasticity. In 2001, Meier et al. (2001) demonstrated the lateral diffusion of the GlyR at the cell surface *via* the use of 500 nm latex beads. Additionally, they confirmed GlyR diffusion alternated between diffusive and confined states, with confinement spatially associated with the scaffold protein gephyrin. This led them to propose a dynamic equilibrium between pools of stabilized and freely mobile receptors (Figure 1). This lateral diffusion was then directly demonstrated *via* the tracking of quantum dots (QDs) bound to surface GlyRs (Dahan et al., 2003). This lateral movement from extrasynaptic pools and switching from free to confined Brownian motion has since been generalized for most neurotransmitter receptors (Thomas et al., 2005; Bogdanov et al., 2006; Pooler and McIlhinney, 2007; Lévi et al., 2008; Bannai et al., 2009; Choquet, 2010; Renner et al., 2017). Differences in diffusion of receptors at extrasynaptic and synaptic sites vary up to 10-fold, as shown for the GABA_A_R (Bannai et al., 2009; de Luca et al., 2017; Hannan et al., 2019) and the GlyR (Dahan et al., 2003; Lévi et al., 2008; Calamai et al., 2009). The characteristic time for receptor exchange by lateral receptor movement is much faster than that related to receptor recycling from internal stores or *de novo* receptor synthesis (Renner et al., 2008).

### Multiple Factors Influence Receptor Diffusion

At the postsynaptic membrane, there are multiple aspects that may influence receptor lateral diffusion. The transient trapping at synapses of laterally diffusing molecules can result from interactions of receptors with other proteins at the membrane such as scaffold molecules, acting as diffusion traps, or from non-specific obstacles, such as molecular crowding, lipid composition and the sub-membrane cytoskeleton (Figure 1).

Interaction of receptors with scaffold molecules represents one of the primary effectors of synaptic diffusion. At the inhibitory synapse, gephyrin interactions have been analyzed for their influence on GABA_A_R (e.g., Jacob et al., 2005; Petrini et al., 2014) and GlyR (e.g., Meier et al., 2001; Meier and Grantyn, 2004) mobility. GlyRs and GABA_A_Rs diffuse far more freely at extrasynaptic sites than when confined in inhibitory synapses at gephyrin clusters. Specifically, gephyrin interaction with receptors at synapses causes transient receptor retention (Meier et al., 2001; Dahan et al., 2003; Calamai et al., 2009; Specht et al., 2011). Furthermore, the binding of the GABA_A_R to gephyrin and subsequent increased dwell time of GABA_A_R at gephyrin-positive synaptic sites affected the synaptic strength of inhibition (Mukherjee et al., 2011). A comparable decrease in diffusion of metabotropic- and AMPA-type glutamate receptors upon binding to their respective scaffold molecules has also been observed (Borgdorff and Choquet, 2002; Sergé et al., 2002).

Competition between receptors, including their subunit composition, may further regulate lateral movement and accumulation into synapses. Lateral diffusion of GABA_A_Rs containing α5 or α2 subunits were reported to be modulated by GABA_B_Rs for binding to scaffold proteins (Gerrow and Triller, 2014). It was recently shown that GABA_A_Rs comprised of different subunit combinations have variable diffusion and synaptic retention rates (Hannan et al., 2019). Additional regulation of receptor diffusion may hence be inferred through subunit-specific regulations, leading to coordinated molecular and functional specificity. Likewise, different diffusion properties arise from contrasting affinities of GABA_A_R and GlyR subunits for gephyrin (Tretter et al., 2008; Maric et al., 2011; Kowalczyk et al., 2013). Finally, the multivalency of the gephyrin scaffold network is also likely to further regulate the molecular organization and diffusion of receptors at the membrane (Specht et al., 2013).

Physical barriers such as cholesterol, phospholipids, other receptors and the cytoskeleton can also regulate the diffusion-trap mechanism. The physical properties of the plasma membrane, including surface geometry, curvature and viscosity determine the flux of receptors (for review, see Marguet et al., 2006). Lipid raft domains can reduce lateral mobility of receptors (Allen et al., 2007), while cholesterol depletion affects apparent membrane viscosity and subsequently receptor diffusion properties (Renner et al., 2009). Thus controlling membrane lipid composition, including cholesterol, can have consequences on molecular flow in and out of the postsynapse. Furthermore, phospholipids within the membrane themselves can act as local messengers in neurotransmission (García-Morales et al., 2015). The tuning of GlyR lateral diffusion has been shown at synaptic and extrasynaptic sites upon F-actin and microtubule disruption respectively (Charrier et al., 2006). Increased lateral diffusion upon dissociation of GABA_A_Rs from their actin anchor, radixin, lead to increased synaptic expression (Loebrich et al., 2006; Hausrat et al., 2015). Additionally, gephyrin interacts with actin filaments *via* several proteins including profilin, Mena/Vasp (Mammoto et al., 1998; Giesemann et al., 2003). Thus the regulation of scaffold trafficking by the cytoskeleton can also affect receptor lateral diffusion and synapse accumulation. Furthermore, activity-dependent extracellular matrix (ECM) modifications may also have structural and functional consequences on receptor lateral mobility (Dityatev et al., 2010). In fact, the secreted ECM molecule Reelin has been shown to regulate the surface distribution and diffusion of NMDA receptors in hippocampal neurons (Groc et al., 2007). The ECM protein thrombospondin-1 increased the lateral diffusion and endocytosis of AMPARs and increased synaptic accumulation of GlyRs in rat spinal cord neurons (Hennekinne et al., 2013). These effects on GlyRs are also dependent on increased excitation as well as the presence of β-1 integrins. Gephyrin clustering itself has been shown to be tuned by integrin-mediated interactions leading to GlyR trapping at the synapse (Charrier et al., 2010). Consequently, the presence of other trans-membrane proteins, in particular those involved in ECM binding such as integrins, can affect neurotransmitter diffusion and synaptic trapping.

Hence surface availability is governed by a combination of processes, such as receptor exocytosis, lateral mobility, diffusion-trapping, dynamic interactions with membrane components, molecular crowding.

## Analyzing Receptor Diffusion Dynamics

Several approaches have been utilized with the aim of quantifying molecular-scale dynamics in cells. Single-molecule fluorescence imaging *via* low-density antibody labeling of GluR2-containing AMPARs enabled visualization of receptor entry and exit at synapses (Tardin et al., 2003). Advances in super-resolution imaging techniques, such as single-particle tracking (SPT) using QDs or coupled with photoactivated localization microscopy (PALM), or universal point accumulation for imaging in nanoscale topography (uPAINT) have enabled further analysis within synapses. uPAINT relies on the binding of fluorescently-labeled ligands or dye-coupled antibodies to the molecule of interest (Sharonov and Hochstrasser, 2006; Giannone et al., 2010). SPT using QDs relies on QD-conjugated antibodies, whereas sptPALM relies on endogenous expression of fluorescent proteins tagged to the target molecule. SPT and uPAINT techniques produce thousands of trajectories generating dense diffusion information with high spatiotemporal resolution. Although it depends on the length of the trajectories, this enables differentiation of active, confined or random movements. These movements can be followed on the cell surface and the landscape of the diffusion dynamics mapped. The most common parameters calculated from these techniques include the diffusion coefficient (*D*) reflecting the area explored and the mean squared displacement (MSD), the function (*f*_(t)_) of which, describes the diffusion behavior over time i.e., Brownian, confined or directed (e.g., Kusumi et al., 1993; Saxton and Jacobson, 1997). QDs bound to receptors and their diffusion provided the first direct demonstration that receptors enter and exit the postsynapse *via* lateral diffusion (Dahan et al., 2003). Although they blink, QD fluorescence is more stable than that of conventional fluorophores with an average size of ~10–15 nm (or bigger if one includes the binding components). Multiple exchanges of GlyRs between extrasynaptic and synaptic domains were observed, with free and confined states respectively. GlyRs were also tracked from one synaptic site to another 4–5 μm away demonstrating synaptic exchange by lateral diffusion of receptors. The *D* was ~0.1 μm^2^/s outside of the synapses, matching that expected for free Brownian diffusion in a lipid bilayer. The *D* then decreased to ~0.02 μm^2^/s (or lower) as it entered the synapse, corresponding to confined movement. QD tracking allows for relatively long acquisition times, yielding long trajectories where changes in diffusion properties can be mapped, however labeling density is low. In comparison, sptPALM, due to the fast bleaching of the fluorophores used, produces much shorter trajectories, but in far larger numbers. Thus, multiple-target tracing (MTT) has been employed to reconnect the single-molecule trajectories and extract their molecular dynamics (Sergé et al., 2008).

Recently, methods to analyze the movement of single molecule trajectories have been advanced with the aim to describe more accurately kinetics of individual interactions in native cell environments. Measuring the *D* of a whole trajectory does not take into account transient stabilizations *via* interactions with other molecules at given locations. Instead, the localized effective binding energy is more ideally suited to analyzing such biochemical interactions (Masson et al., 2014). Therefore, Masson et al. (2014) suggested an approach using Bayesian inference and overdamped Langevin equations to analyze the molecular motion. This generates an energy landscape which takes into account the heterogeneous diffusivity in the cell membrane. The depth of the energy trap is modulated by biochemical interactions between the receptor and scaffold proteins (Masson et al., 2014). This spatial cartography demonstrated that the presence of gephyrin clusters coincided with energy minima and hence was consistent with transient stabilization of receptors at synapses (El Beheiry et al., 2015). Consequently, the neuronal membrane has to be considered as a statistical field with constantly moving and transiently trapped molecules rather than formed by the juxtaposition of domains with fixed compositions. Using computer simulations and mathematical modeling, insight into molecular dynamics and the relationship between transient receptor trapping and local chemical reactions has been estimated. Moreover, using a Markovian approach, synaptic weight could be expressed as fluctuations in the number of bound receptors in the postsynapse (Holcman and Triller, 2006). Langevin equation models have enabled an analysis of molecular interactions of AMPARs (Hoze et al., 2012) and GlyRs (Masson et al., 2014; El Beheiry et al., 2015) in the postsynaptic membrane. Additionally, the geometrical effect of membrane curvature on the 2D projected stochastic trajectory of a molecule affects diffusion properties (Domanov et al., 2011; Renner et al., 2011). The introduction of another parameter different from MTT based tracking and reconnection, the packing coefficient (Pc), allows characterization of the movement of a given molecule along its trajectory, thus as a function of time, independently of its overall diffusivity (Renner et al., 2017). It can also be utilized to derivate the effective *K*_on_ and *K*_off_ of a receptor to its scaffold. A cooperative mesoscopic model of the reciprocal stabilization of synaptic receptors and scaffolding proteins allowed accounting for the synapse “stability” as a quasi-equilibrium (Sekimoto and Triller, 2009). Then, using an out-of-equilibrium model, it has been proposed that the size of the scaffold clusters can be explained by the aggregation of gephyrin proteins diffusing in the sub-membrane space whilst bound to the GlyR, balanced against membrane turnover “aggregation-removal model” (Ranft et al., 2017).

The combination of theoretical modeling with single-molecule experimental data can now provide a quantification of synapse receptor dynamics in relation to the chemical modulation of these dynamics in the live cell environment, thus the concept of “chemistry *in-cellulo*” (Salvatico et al., 2015). This mixed experimental and theoretical approach will enable comprehension of how the dynamic movement of receptors and their interactions with other proteins can lead to longer-term stabilizations as well as the chemical determinants of receptor number and synapse function.

## Activity Regulation of Inhibitory Receptor Diffusion

Many studies in recent years have concentrated on molecular mechanisms of inhibitory neurotransmission and synaptic scaffold protein modification that influence the local interactions and diffusion events underlying synaptic plasticity. It is now established that receptor diffusion-trapping at synapses can be affected by neuronal activity (Figure 1). Increased mobility of QD-labeled GlyRs and GABA_A_Rs has been seen upon increased excitatory neuronal activity (Lévi et al., 2008; Bannai et al., 2009; Muir et al., 2010). Application of TTX to spinal cord neurons demonstrated reduced lateral diffusion of GlyR with an increase in receptor cluster number, but not for GABA_A_Rs (Lévi et al., 2008). However the same effect was not seen for GABA_A_Rs in hippocampal cells, instead, TTX application reduced GABA_A_R lateral diffusion by means of an NMDA-calcineurin-dependent mechanism (Bannai et al., 2009), suggesting cell type and receptor type plays an additional regulatory role. In fact the involvement of NMDARs in the exocytosis of GABA_A_Rs is CaMKII-dependent, and consequently potentiates inhibitory transmission (iLTP) (Marsden et al., 2007). It should be noted NMDAR-induced iLTP leads to a moderate intracellular calcium recruitment and activation of CaMKII (Lucchesi et al., 2011; Petrini et al., 2014). Conversely, iLTD *via* NMDARs and voltage-gated calcium channels leads to a massive increase in intracellular calcium and subsequent recruitment of calcineurin to inhibitory synapses (Bannai et al., 2009; Muir et al., 2010). These converging pathways constitute a fine-tuning of activity-dependent GABA_A_R diffusion dynamics and thus inhibition. More precisely, calcineurin-induced phosphorylation of GABA_A_Rs following NMDA activation confirmed the GABA_A_R dispersal with important implications for activity-dependent control of synaptic inhibition (Muir et al., 2010). Conversely, GABA_A_R cluster promotion at the postsynapse and enhanced GABAergic signaling *via* a metabotropic glutamate receptor-induced IP3 and PKC signaling pathway show spatiotemporal signaling patterns of calcium can fine-tune GABA_A_R availability (Bannai et al., 2015). As shown in spinal cord neurons, PKC also phosphorylates the GlyR β-subunit at residue S403 (Specht et al., 2011). Consequently cross talk and competition between GABA_A_Rs and GlyRs, at mixed GABA-Gly synapses in the spinal cord, adds an additional layer of complexity to the regulation of synaptic inhibition. Combining experimental work and theory it has been hypothesized that the long-term stability of synaptic cluster size obeys a dynamic equilibrium between the attraction of scaffold molecules to each other and the repulsion of receptor-receptor interactions (Haselwandter et al., 2011). Other synaptic components also impact these interactions. Upon chemical iLTP, GABA_A_Rs are immobilized at synapses following active gephyrin recruitment in hippocampal neurons, the mechanism of which requires phosphorylation of GABA_A_R-β3 by CaMKIIα (Petrini et al., 2014). Whilst gephyrin plays a critical role in GABA_A_R membrane clustering, gephyrin-independent mechanisms of GABA_A_R stabilization also exist. Following sustained excitatory activity GABA_A_R mobility and clustering was shown to be independent of gephyrin clustering in hippocampal neurons (Niwa et al., 2012). More recently, QD-SPT combined with optogenetics to control calcium flow with high temporal precision showed inter-synaptic lateral diffusion of GABA_A_Rs in a desensitized state in hippocampal neurons (de Luca et al., 2017). Synapses were typically 2–4 μm apart, with intersynaptic diffusion occurring in ~15% trajectories at a *D* of 0.07 μm^2^S^−1^. Further, they showed that glutamatergic activity limits this inter-synaptic diffusion *via* trapping GABA_A_Rs at excitatory synapses. They suggested this might present a mechanism by which a memory of recent activation is transmitted to neighboring synapses. In addition to regulation of inhibitory synaptic receptors *via* direct neuronal activity, microglia have also been implicated in receptor dynamics. Prostaglandin E2 from microglia was recently shown to regulate GlyR diffusion dynamics and synaptic trapping but not GABAergic synapses (Cantaut-Belarif et al., 2017). Importantly, this demonstrated that microglia could regulate the plasticity of glycinergic synapses by tuning GlyR diffusion-trapping. Hence diffusion-trapping is not a cell-autonomous event. Additional fine-tuning of receptor diffusion dynamics may further occur in certain inflammatory states.

Recent work into the organization within synaptic clusters of receptor proteins and scaffold molecules have revealed the existence of subsynaptic domains in both excitatory (e.g., MacGillavry et al., 2013; Nair et al., 2013) and inhibitory (e.g., Specht et al., 2013; Crosby et al., 2019) synapses. In spinal cord neurons the stoichiometry of gephyrin to GlyR binding sites was estimated to be approximately 1:1 (Specht et al., 2013). Incorporating super-resolution microscopy and model simulations, gephyrin stabilization in nano-domains was visualized upon iLTP which in turn stabilized the number of GABA_A_Rs in mouse hippocampal neurons (Pennacchietti et al., 2017). In a separate study, QD-SPT of GABA_A_R diffusion in rat hippocampal neurons showed GSK-3β and ERK1/2 differentially altered the gephyrin scaffold mesh, which as a result affected GABA_A_R surface dynamics (Battaglia et al., 2018). They found that gephyrin microdomain compaction was regulated by phosphorylation in an activity-dependent way. Future work into this nano-organization and its control on intrasynaptic diffusion will allow understanding of long-term synaptic stability and GABA_A_R/GlyR competition at inhibitory synapses.

## Functional Consequences of Diffusion Regulation

Diffusion trapping of receptors at synapses tunes receptor number, hence regulating neuronal activity with functional consequences on synaptic plasticity (Choquet and Triller, 2013; Petrini and Barberis, 2014). Plasticity associated changes in lateral mobility have been shown in inhibitory (e.g., Bannai et al., 2009; Petrini et al., 2014) and excitatory synapses (e.g., Ehlers et al., 2007; Makino and Malinow, 2009). In one such example, tracking surface GABA_A_Rs on cultured hippocampal neurons during chemical iLTP showed synaptic recruitment of gephyrin from extrasynaptic regions was promoted by CamKII-dependent phosphorylation of GABA_A_R-β3 at Ser838 (Petrini et al., 2014). Further, they showed that impairment of gephyrin assembly prevented chemical iLTP with an associated decrease in GABA_A_R immobilization at synapses. Concurrently, changes in the exocytosis of inhibitory receptors can also occur upon neuronal activation, but over slower time courses (Marsden et al., 2007). This activity-dependent plasticity is hence determined by diffusion of the molecular synaptic components and the underlying mechanisms that regulate receptor availability across multi-time scales.

The link between lateral diffusion of receptors and their confinement at synapses with behavior is not yet understood. However, one mechanism has recently been described, linking RhoA/ROCK activity-dependent phosphorylation of radixin which in turn uncouples GABA_A_R-α5 from extrasynaptic sites enabling their enrichment at synapses (Hausrat et al., 2015). This radixin phosphorylation was shown to occur in wild-type mice during short-term memory and reversal learning. In excitatory synapses, interfering with AMPAR surface diffusion impaired synaptic potentiation of Schaffer collaterals and commissural inputs to the CA1 of the mouse hippocampus in cultured slices and *in vivo* (Penn et al., 2017). Moreover, they showed AMPAR immobilization in the hippocampus *in vivo* inhibited fear conditioning. Thus, lateral diffusion of receptors and their temporal confinement at both excitatory and inhibitory synapses is likely to be a fundamental mechanism involved in learning and memory.

Affecting local and network-wide activity, diffusion dynamics may be implicated in certain neuropathologies. Benzodiazepines (BZDs) are widely used to treat many neurological and psychiatric diseases. It is now thought that in addition to their effects on receptor gating, membrane dynamics are also affected. SPT experiments of GABA_A_Rs in mouse hippocampal neurons upon addition of the GABA_A_R agonist muscimol showed accelerated GABA_A_R diffusion, which was subsequently abolished upon addition of the BZD agonist diazepam (Gouzer et al., 2014). Using SPT in hippocampal neurons, diazepam was shown to increase synaptic stabilization and clustering of GABA_A_Rs and decreased their lateral diffusion upon sustained neuronal activity but not at rest (Gouzer et al., 2014; Lévi et al., 2015). Acute estradiol treatment has also been demonstrated to decrease the confinement of GABA_A_Rs, reducing their dwell time in synaptic compartments and increasing the *D* at extrasynaptic sites (Mukherjee et al., 2017). These results have a direct impact on the design of therapeutic compounds for diseases arising from dysregulation of inhibition.

## Conclusions and Perspectives

The plasma membrane is dynamic and trans-membrane molecules such as receptors diffuse laterally. These processes provide mechanisms for regulation of receptor number at synapses and thus function and plasticity. Recent results have isolated various pathways involved in receptor diffusion control, however, there are many important questions still to be answered. The contribution of receptor dynamics in synapse development, maturation, and refinement in both health and disease is yet to be fully explored. The interplay of GABA_A_R and GlyR competition within inhibitory synapses in different brain regions, alongside distance and distribution of inhibitory and excitatory receptors is likely to underpin activity-dependent modification of synapse strength.

Whilst there have been huge technological advancements over a relatively short period of time, there remains inherent limitations in the currently used techniques for analyzing lateral membrane diffusion. QDs are a popular choice due to their photostability, bright fluorescence, long trajectories and ability to multiplex (Cutler et al., 2013; Kakizuka et al., 2016; Renner et al., 2017). However, their large size complexed with antibodies can sterically hinder lateral mobility (Abraham et al., 2017) and low-density labeling strategies mean only a fraction of the molecules are probed. The use of sptPALM enables direct genetic tagging of target molecules with a fluorescent protein, either by lentiviral expression or knock-in animal models, allowing tracking of all target molecules and analysis of endogenous molecule copy number (Lee et al., 2012; Specht et al., 2013). However incorrect protein folding and targeted degradation of the fluorescent protein-target complex can occur (Tanudji et al., 2002; Stepanenko et al., 2013; Guo et al., 2014) and trajectories are shorter than those of QDs. uPAINT relies on binding of high affinity fluorescently tagged antibodies or ligands to the target (Giannone et al., 2010). The main drawback of this technique is the saturation of the target with bleached ligands. Future technical developments will include the manipulation of fluorescent proteins and organic probes to be smaller and brighter, improvements in microscope set-ups to track multiple proteins simultaneously, improved resolution in 3D imaging and tracking, and use of brain slices and *in vivo* set-ups will provide additional comprehension of diffusion dynamics within a biologically relevant microenvironment.

## Author Contributions

All authors listed have made a substantial, direct and intellectual contribution to the work, and approved it for publication.

## Conflict of Interest

The authors declare that the research was conducted in the absence of any commercial or financial relationships that could be construed as a potential conflict of interest. The handling Editor declared a past co-authorship with one of the authors AT.

## References

[B1] Abraham L. Lu H. Y. Falcão R. C. Scurll J. Jou T. Irwin B.. (2017). Limitations of Qdot labelling compared to directly-conjugated probes for single particle tracking of B cell receptor mobility. Sci. Rep. 7:11379. 10.1038/s41598-017-11563-9 28900238 PMC5595841

[B2] Allen J. A. Halverson-Tamboli R. A. Rasenick M. M. (2007). Lipid raft microdomains and neurotransmitter signalling. Nat. Rev. Neurosci. 8, 128–140. 10.1038/nrn2059 17195035

[B3] Bannai H. Lévi S. Schweizer C. Inoue T. Launey T. Racine V.. (2009). Activity-dependent tuning of inhibitory neurotransmission based on GABA_A_R diffusion dynamics. Neuron 62, 670–682. 10.1016/j.neuron.2009.04.023 19524526

[B4] Bannai H. Niwa F. Sherwood M. W. Shrivastava A. N. Arizono M. Miyamoto A.. (2015). Bidirectional control of synaptic GABA_A_R clustering by glutamate and calcium. Cell Rep. 13, 2768–2780. 10.1016/j.celrep.2015.12.002 26711343 PMC4700050

[B5] Battaglia S. Renner M. Russeau M. Côme E. Tyagarajan S. K. Lévi S. (2018). Activity-dependent inhibitory synapse scaling is determined by gephyrin phosphorylation and subsequent regulation of GABA_A_ receptor diffusion. eNeuro 5:ENEURO.0203-17.2017. 10.1523/eneuro.0203-17.2017 29379879 PMC5780843

[B6] Bogdanov Y. Michels G. Armstrong-Gold C. Haydon P. G. Lindstrom J. Pangalos M.. (2006). Synaptic GABA_A_ receptors are directly recruited from their extrasynaptic counterparts. EMBO J. 25, 4381–4389. 10.1038/sj.emboj.7601309 16946701 PMC1570424

[B7] Borgdorff A. J. Choquet D. (2002). Regulation of AMPA receptor lateral movements. Nature 417, 649–653. 10.1038/nature00780 12050666

[B8] Calamai M. Specht C. G. Heller J. Alcor D. Machado P. Vannier C.. (2009). Gephyrin oligomerization controls GlyR mobility and synaptic clustering. J. Neurosci. 29, 7639–7648. 10.1523/JNEUROSCI.5711-08.2009 19535575 PMC6665613

[B9] Cantaut-Belarif Y. Antri M. Pizzarelli R. Colasse S. Vaccari I. Soares S.. (2017). Microglia control the glycinergic but not the GABAergic synapses *via* prostaglandin E2 in the spinal cord. J. Cell Biol. 216, 2979–2989. 10.1083/jcb.201607048 28716844 PMC5584146

[B10] Charrier C. Ehrensperger M.-V. Dahan M. Lévi S. Triller A. (2006). Cytoskeleton regulation of glycine receptor number at synapses and diffusion in the plasma membrane. J. Neurosci. 26, 8502–8511. 10.1523/JNEUROSCI.1758-06.2006 16914675 PMC6674337

[B11] Charrier C. Machado P. Tweedie-Cullen R. Y. Rutishauser D. Mansuy I. M. Triller A. (2010). A crosstalk between β1 and β3 integrins controls glycine receptor and gephyrin trafficking at synapses. Nat. Neurosci. 13, 1388–1395. 10.1038/nn.2645 20935643

[B12] Choquet D. (2010). Fast AMPAR trafficking for a high-frequency synaptic transmission. Eur. J. Neurosci. 32, 250–260. 10.1111/j.1460-9568.2010.07350.x 20646044

[B13] Choquet D. Triller A. (2013). The dynamic synapse. Neuron 80, 691–703. 10.1016/j.neuron.2013.10.013 24183020

[B14] Crick F. (1984). Neurobiology: memory and molecular turnover. Nature 312:101. 10.1038/312101a0 6504122

[B15] Crosby K. C. Gookin S. E. Garcia J. D. Hahm K. M. Dell’Acqua M. L. Smith K. R. (2019). Nanoscale subsynaptic domains underlie the organization of the inhibitory synapse. Cell Rep. 26, 3284.e3–3297.e3. 10.1016/j.celrep.2019.02.070 30893601 PMC6529211

[B16] Cutler P. J. Malik M. D. Liu S. Byars J. M. Lidke D. S. Lidke K. A. (2013). Multi-color quantum dot tracking using a high-speed hyperspectral line-scanning microscope. PLoS One 8:e64320. 10.1371/journal.pone.0064320 23717596 PMC3661486

[B17] Dahan M. Lévi S. Luccardini C. Rostaing P. Riveau B. Triller A. (2003). Diffusion dynamics of glycine receptors revealed by single-quantum dot tracking. Science 302, 442–445. 10.1126/science.1088525 14564008

[B18] de Luca E. Ravasenga T. Petrini E. M. Polenghi A. Nieus T. Guazzi S.. (2017). Inter-synaptic lateral diffusion of GABA_A_ receptors shapes inhibitory synaptic currents. Neuron 95, 63.e5–69.e5. 10.1016/j.neuron.2017.06.022 28683270 PMC5500312

[B19] Dityatev A. Schachner M. Sonderegger P. (2010). The dual role of the extracellular matrix in synaptic plasticity and homeostasis. Nat. Rev. Neurosci. 11, 735–746. 10.1038/nrn2898 20944663

[B20] Domanov Y. A. Aimon S. Toombes G. E. S. Renner M. Quemeneur F. Triller A.. (2011). Mobility in geometrically confined membranes. Proc. Natl. Acad. Sci. U S A 108, 12605–12610. 10.1073/pnas.1102646108 21768336 PMC3150897

[B21] Ehlers M. D. Heine M. Groc L. Lee M.-C. Choquet D. (2007). Diffusional trapping of GluR1 AMPA receptors by input-specific synaptic activity. Neuron 54, 447–460. 10.1016/j.neuron.2007.04.010 17481397 PMC1993808

[B22] El Beheiry M. Dahan M. Masson J.-B. (2015). InferenceMAP: mapping of single-molecule dynamics with Bayesian inference. Nat. Methods 12, 594–595. 10.1038/nmeth.3441 26125589

[B23] Fujii S. Tanaka H. Hirano T. (2018). Suppression of AMPA receptor exocytosis contributes to hippocampal LTD. J. Neurosci. 38, 5523–5537. 10.1523/JNEUROSCI.3210-17.2018 29899033 PMC8174134

[B24] García-Morales V. Montero F. González-Forero D. Rodríguez-Bey G. Gómez-Pérez L. Medialdea-Wandossell M. J.. (2015). Membrane-derived phospholipids control synaptic neurotransmission and plasticity. PLoS Biol. 13:e1002153. 10.1371/journal.pbio.1002153 25996636 PMC4440815

[B25] Gerrow K. Triller A. (2014). GABA_A_ receptor subunit composition and competition at synapses are tuned by GABAB receptor activity. Mol. Cell. Neurosci. 60, 97–107. 10.1016/j.mcn.2014.04.001 24747870

[B26] Giannone G. Hosy E. Levet F. Constals A. Schulze K. Sobolevsky A. I.. (2010). Dynamic superresolution imaging of endogenous proteins on living cells at ultra-high density. Biophys. J. 99, 1303–1310. 10.1016/j.bpj.2010.06.005 20713016 PMC2920718

[B27] Giesemann T. Schwarz G. Nawrotzki R. Berhörster K. Rothkegel M. Schlüter K.. (2003). Complex formation between the postsynaptic scaffolding protein gephyrin, profilin, and Mena: a possible link to the microfilament system. J. Neurosci. 23, 8330–8339. 10.1523/JNEUROSCI.23-23-08330.2003 12967995 PMC6740687

[B28] Gouzer G. Specht C. G. Allain L. Shinoe T. Triller A. (2014). Benzodiazepine-dependent stabilization of GABA_A_ receptors at synapses. Mol. Cell. Neurosci. 63, 101–113. 10.1016/j.mcn.2014.10.004 25466558

[B29] Groc L. Choquet D. Stephenson F. A. Verrier D. Manzoni O. J. Chavis P. (2007). NMDA receptor surface trafficking and synaptic subunit composition are developmentally regulated by the extracellular matrix protein reelin. J. Neurosci. 27, 10165–10175. 10.1523/JNEUROSCI.1772-07.2007 17881522 PMC6672660

[B30] Guo M. Gelman H. Gruebele M. (2014). Coupled protein diffusion and folding in the cell. PLoS One 9:e113040. 10.1371/journal.pone.0113040 25436502 PMC4249841

[B31] Hannan S. Minere M. Harris J. Izquierdo P. Thomas P. Tench B.. (2019). GABA_A_R isoform and subunit structural motifs determine synaptic and extrasynaptic receptor localisation. Neuropharmacology [Epub ahead of print]. 10.1016/j.neuropharm.2019.02.022 30794836

[B32] Haselwandter C. A. Calamai M. Kardar M. Triller A. da Silveira R. A. (2011). Formation and stability of synaptic receptor domains. Phys. Rev. Lett. 106:238104. 10.1103/physrevlett.106.238104 21770547

[B33] Hausrat T. J. Muhia M. Gerrow K. Thomas P. Hirdes W. Tsukita S.. (2015). Radixin regulates synaptic GABA_A_ receptor density and is essential for reversal learning and short-term memory. Nat. Commun. 6:6872. 10.1038/ncomms7872 25891999 PMC4411296

[B34] Hayashi Y. Shi S. H. Esteban J. A. Piccini A. Poncer J. C. Malinow R. (2000). Driving AMPA receptors into synapses by LTP and CaMKII: requirement for GluR1 and PDZ domain interaction. Science 287, 2262–2267. 10.1126/science.287.5461.2262 10731148

[B35] Hennekinne L. Colasse S. Triller A. Renner M. (2013). Differential control of thrombospondin over synaptic glycine and AMPA receptors in spinal cord neurons. J. Neurosci. 33, 11432–11439. 10.1523/JNEUROSCI.5247-12.2013 23843515 PMC6618694

[B36] Holcman D. Triller A. (2006). Modeling synaptic dynamics driven by receptor lateral diffusion. Biophys. J. 91, 2405–2415. 10.1529/biophysj.106.081935 16844759 PMC1562376

[B37] Hoze N. Nair D. Hosy E. Sieben C. Manley S. Herrmann A.. (2012). Heterogeneity of AMPA receptor trafficking and molecular interactions revealed by superresolution analysis of live cell imaging. Proc. Natl. Acad. Sci. U S A 109, 17052–17057. 10.1073/pnas.1204589109 23035245 PMC3479500

[B38] Jacob T. C. Bogdanov Y. D. Magnus C. Saliba R. S. Kittler J. T. Haydon P. G.. (2005). Gephyrin regulates the cell surface dynamics of synaptic GABA_A_ receptors. J. Neurosci. 25, 10469–10478. 10.1523/JNEUROSCI.2267-05.2005 16280585 PMC6725824

[B39] Kakizuka T. Ikezaki K. Kaneshiro J. Fujita H. Watanabe T. M. Ichimura T. (2016). Simultaneous nano-tracking of multiple motor proteins *via* spectral discrimination of quantum dots. Biomed. Opt. Express 7, 2475–2493. 10.1364/boe.7.002475 27446684 PMC4948608

[B40] Kittler J. T. Delmas P. Jovanovic J. N. Brown D. A. Smart T. G. Moss S. J. (2000). Constitutive endocytosis of GABA_A_ receptors by an association with the adaptin AP2 complex modulates inhibitory synaptic currents in hippocampal neurons. J. Neurosci. 20, 7972–7977. 10.1523/JNEUROSCI.20-21-07972.2000 11050117 PMC6772725

[B41] Kowalczyk S. Winkelmann A. Smolinsky B. Förstera B. Neundorf I. Schwarz G.. (2013). Direct binding of GABA_A_ receptor β2 and β3 subunits to gephyrin. Eur. J. Neurosci. 37, 544–554. 10.1111/ejn.12078 23205938

[B42] Kusumi A. Sako Y. Yamamoto M. (1993). Confined lateral diffusion of membrane receptors as studied by single particle tracking (nanovid microscopy). Effects of calcium-induced differentiation in cultured epithelial cells. Biophys. J. 65, 2021–2040. 10.1016/s0006-3495(93)81253-0 8298032 PMC1225938

[B43] Lee S.-H. Shin J. Y. Lee A. Bustamante C. (2012). Counting single photoactivatable fluorescent molecules by photoactivated localization microscopy (PALM). Proc. Natl. Acad. Sci. U S A 109, 17436–17441. 10.1073/pnas.1215175109 23045631 PMC3491528

[B44] Lévi S. Le Roux N. Eugène E. Poncer J. C. (2015). Benzodiazepine ligands rapidly influence GABA_A_ receptor diffusion and clustering at hippocampal inhibitory synapses. Neuropharmacology 88, 199–208. 10.1016/j.neuropharm.2014.06.002 24930360

[B45] Lévi S. Schweizer C. Bannai H. Pascual O. Charrier C. Triller A. (2008). Homeostatic regulation of synaptic GlyR numbers driven by lateral diffusion. Neuron 59, 261–273. 10.1016/j.neuron.2008.05.030 18667154

[B46] Loebrich S. Bähring R. Katsuno T. Tsukita S. Kneussel M. (2006). Activated radixin is essential for GABA_A_ receptor α5 subunit anchoring at the actin cytoskeleton. EMBO J. 25, 987–999. 10.1038/sj.emboj.7600995 16467845 PMC1409722

[B47] Lucchesi W. Mizuno K. Giese K. P. (2011). Novel insights into CaMKII function and regulation during memory formation. Brain Res. Bull. 85, 2–8. 10.1016/j.brainresbull.2010.10.009 21070840

[B48] Lüthi A. Chittajallu R. Duprat F. Palmer M. J. Benke T. A. Kidd F. L.. (1999). Hippocampal LTD expression involves a pool of AMPARs regulated by the NSF-GluR2 interaction. Neuron 24, 389–399. 10.1016/s0896-6273(00)80852-1 10571232

[B49] MacGillavry H. D. Song Y. Raghavachari S. Blanpied T. A. (2013). Nanoscale scaffolding domains within the postsynaptic density concentrate synaptic AMPA receptors. Neuron 78, 615–622. 10.1016/j.neuron.2013.03.009 23719161 PMC3668352

[B50] Makino H. Malinow R. (2009). AMPA receptor incorporation into synapses during LTP: the role of lateral movement and exocytosis. Neuron 64, 381–390. 10.1016/j.neuron.2009.08.035 19914186 PMC2999463

[B51] Mammen A. Huganir R. O’Brien R.-J. (1997). Redistribution and stabilization of cell surface glutamate receptors during synapse formation. J. Neurosci. 17, 7351–7358. 10.1523/JNEUROSCI.17-19-07351.1997 9295381 PMC6573457

[B52] Mammoto A. Sasaki T. Asakura T. Hotta I. Imamura H. Takahashi K.. (1998). Interactions of drebrin and gephyrin with profilin. Biochem. Biophys. Res. Commun. 243, 86–89. 10.1006/bbrc.1997.8068 9473484

[B53] Marguet D. Lenne P.-F. Rigneault H. He H.-T. (2006). Dynamics in the plasma membrane: how to combine fluidity and order. EMBO J. 25, 3446–3457. 10.1038/sj.emboj.7601204 16900097 PMC1538569

[B54] Maric H.-M. Mukherjee J. Tretter V. Moss S. J. Schindelin H. (2011). Gephyrin-mediated γ-aminobutyric acid type A and glycine receptor clustering relies on a common binding site. J. Biol. Chem. 286, 42105–42114. 10.1074/jbc.M111.303412 22006921 PMC3234978

[B55] Marsden K. C. Beattie J. B. Friedenthal J. Carroll R. C. (2007). NMDA receptor activation potentiates inhibitory transmission through GABA receptor-associated protein-dependent exocytosis of GABA_A_ receptors. J. Neurosci. 27, 14326–14337. 10.1523/JNEUROSCI.4433-07.2007 18160640 PMC6673443

[B56] Masson J.-B. Dionne P. Salvatico C. Renner M. Specht C. G. Triller A.. (2014). Mapping the energy and diffusion landscapes of membrane proteins at the cell surface using high-density single-molecule imaging and bayesian inference: application to the multiscale dynamics of glycine receptors in the neuronal membrane. Biophys. J. 106, 74–83. 10.1016/j.bpj.2013.10.027 24411239 PMC3907207

[B57] Meier J. Grantyn R. (2004). A gephyrin-related mechanism restraining glycine receptor anchoring at GABAergic synapses. J. Neurosci. 24, 1398–1405. 10.1523/JNEUROSCI.4260-03.2004 14960612 PMC6730342

[B58] Meier J. Vannier C. Sergé A. Triller A. Choquet D. (2001). Fast and reversible trapping of surface glycine receptors by gephyrin. Nat. Neurosci. 4, 253–260. 10.1038/85099 11224541

[B59] Muir J. Arancibia-Carcamo I. L. MacAskill A. F. Smith K. R. Griffin L. D. Kittler J. T. (2010). NMDA receptors regulate GABA_A_ receptor lateral mobility and clustering at inhibitory synapses through serine 327 on the 2 subunit. Proc. Natl. Acad. Sci. U S A 107, 16679–16684. 10.1073/pnas.1000589107 20823221 PMC2944765

[B60] Mukherjee J. Cardarelli R. A. Cantaut-Belarif Y. Deeb T. Z. Srivastava D. P. Tyagarajan S. K.. (2017). Estradiol modulates the efficacy of synaptic inhibition by decreasing the dwell time of GABA_A_ receptors at inhibitory synapses. Proc. Natl. Acad. Sci. U S A 114, 11763–11768. 10.1073/pnas.1705075114 29078280 PMC5676881

[B61] Mukherjee J. Kretschmannova K. Gouzer G. Maric H.-M. Ramsden S. Tretter V.. (2011). The residence time of GABA_A_Rs at inhibitory synapses is determined by direct binding of the receptor 1 subunit to gephyrin. J. Neurosci. 31, 14677–14687. 10.1523/JNEUROSCI.2001-11.2011 21994384 PMC3202462

[B62] Nair D. Hosy E. Petersen J. D. Constals A. Giannone G. Choquet D.. (2013). Super-resolution imaging reveals that AMPA receptors inside synapses are dynamically organized in nanodomains regulated by PSD95. J. Neurosci. 33, 13204–13224. 10.1523/JNEUROSCI.2381-12.2013 23926273 PMC6619720

[B63] Nishimune A. Isaac J. T. Molnar E. Noel J. Nash S. R. Tagaya M.. (1998). NSF binding to GluR2 regulates synaptic transmission. Neuron 21, 87–97. 10.1016/s0896-6273(00)80517-6 9697854

[B64] Niwa F. Bannai H. Arizono M. Fukatsu K. Triller A. Mikoshiba K. (2012). Gephyrin-independent GABA_A_R mobility and clustering during plasticity. PLoS One 7:e36148 10.1371/journal.pone.0036148 22563445 PMC3338568

[B65] Nusser Z. Hájos N. Somogyi P. Mody I. (1998). Increased number of synaptic GABA_A_ receptors underlies potentiation at hippocampal inhibitory synapses. Nature 395, 172–177. 10.1038/25999 9744275

[B66] Park M. Penick E. C. Edwards J. G. Kauer J. A. Ehlers M. D. (2004). Recycling endosomes supply AMPA receptors for LTP. Science 305, 1972–1975. 10.1126/science.1102026 15448273

[B67] Passafaro M. Piëch V. Sheng M. (2001). Subunit-specific temporal and spatial patterns of AMPA receptor exocytosis in hippocampal neurons. Nat. Neurosci. 4, 917–926. 10.1038/nn0901-917 11528423

[B68] Penn A. C. Zhang C. L. Georges F. Royer L. Breillat C. Hosy E.. (2017). Hippocampal LTP and contextual learning require surface diffusion of AMPA receptors. Nature 549, 384–388. 10.1038/nature23658 28902836 PMC5683353

[B69] Pennacchietti F. Vascon S. Nieus T. Rosillo C. Das S. Tyagarajan S. K.. (2017). Nanoscale molecular reorganization of the inhibitory postsynaptic density is a determinant of GABAergic synaptic potentiation. J. Neurosci. 37, 1747–1756. 10.1523/JNEUROSCI.0514-16.2016 28073939 PMC6589977

[B70] Petrini E. M. Barberis A. (2014). Diffusion dynamics of synaptic molecules during inhibitory postsynaptic plasticity. Front. Cell. Neurosci. 8:300. 10.3389/fncel.2014.00300 25294987 PMC4171989

[B71] Petrini E. M. Ravasenga T. Hausrat T. J. Iurilli G. Olcese U. Racine V.. (2014). Synaptic recruitment of gephyrin regulates surface GABA_A_ receptor dynamics for the expression of inhibitory LTP. Nat. Commun. 5:3921. 10.1038/ncomms4921 24894704 PMC4059940

[B72] Poo M. Cone R. A. (1974). Lateral diffusion of rhodopsin in the photoreceptor membrane. Nature 247, 438–441. 10.1038/247438a0 4818543

[B73] Pooler A. M. McIlhinney R. A. J. (2007). Lateral diffusion of the GABAB receptor is regulated by the GABAB2 C terminus. J. Biol. Chem. 282, 25349–25356. 10.1074/jbc.M702358200 17597070

[B74] Ranft J. Almeida L. G. Rodriguez P. C. Triller A. Hakim V. (2017). An aggregation-removal model for the formation and size determination of post-synaptic scaffold domains. PLoS Comput. Biol. 13:e1005516. 10.1371/journal.pcbi.1005516 28437460 PMC5421815

[B75] Renner M. Choquet D. Triller A. (2009). Control of the postsynaptic membrane viscosity. J. Neurosci. 29, 2926–2937. 10.1523/JNEUROSCI.4445-08.2009 19261888 PMC6666215

[B76] Renner M. Specht C. G. Triller A. (2008). Molecular dynamics of postsynaptic receptors and scaffold proteins. Curr. Opin. Neurobiol. 18, 532–540. 10.1016/j.conb.2008.09.009 18832033

[B200] Renner M. Domanov Y. Sandrin F. Izeddin I. Bassereau P. Triller A. (2011). Lateral diffusion on tubular membranes: quantification of measurements bias. PLoS One 6:e25731. 10.1371/journal.pone.0025731 21980531 PMC3183067

[B77] Renner M. Wang L. Levi S. Hennekinne L. Triller A. (2017). A simple and powerful analysis of lateral subdiffusion using single particle tracking. Biophys. J. 113, 2452–2463. 10.1016/j.bpj.2017.09.017 29211999 PMC5738498

[B78] Rosenberg M. Meier J. Triller A. Vannier C. (2001). Dynamics of glycine receptor insertion in the neuronal plasma membrane. J. Neurosci. 21, 5036–5044. 10.1523/jneurosci.21-14-05036.2001 11438579 PMC6762839

[B79] Salvatico C. Specht C. G. Triller A. (2015). Synaptic receptor dynamics: from theoretical concepts to deep quantification and chemistry in cellulo. Neuropharmacology 88, 2–9. 10.1016/j.neuropharm.2014.09.020 25261785

[B80] Saxton M. J. Jacobson K. (1997). Single-particle tracking: applications to membrane dynamics. Annu. Rev. Biophys. Biomol. Struct. 26, 373–399. 10.1146/annurev.biophys.26.1.373 9241424

[B81] Sekimoto K. Triller A. (2009). Compatibility between itinerant synaptic receptors and stable postsynaptic structure. Phys. Rev. E Stat. Nonlin. Soft Matter Phys. 79:031905. 10.1103/physreve.79.031905 19391969

[B82] Sergé A. Bertaux N. Rigneault H. Marguet D. (2008). Dynamic multiple-target tracing to probe spatiotemporal cartography of cell membranes. Nat. Methods 5, 687–694. 10.1038/nmeth.1233 18604216

[B83] Sergé A. Fourgeaud L. Hémar A. Choquet D. (2002). Receptor activation and homer differentially control the lateral mobility of metabotropic glutamate receptor 5 in the neuronal membrane. J. Neurosci. 22, 3910–3920. 10.1523/jneurosci.22-10-03910.2002 12019310 PMC6757631

[B84] Sharonov A. Hochstrasser R. M. (2006). Wide-field subdiffraction imaging by accumulated binding of diffusing probes. Proc. Natl. Acad. Sci. U S A 103, 18911–18916. 10.1073/pnas.0609643104 17142314 PMC1748151

[B85] Singer S. J. Nicolson G. L. (1972). The fluid mosaic model of the structure of cell membranes. Science 175, 720–731. 10.1126/science.175.4023.720 4333397

[B86] Song I. Huganir R. L. (2002). Regulation of AMPA receptors during synaptic plasticity. Trends Neurosci. 25, 578–588. 10.1016/s0166-2236(02)02270-1 12392933

[B87] Specht C. G. Grünewald N. Pascual O. Rostgaard N. Schwarz G. Triller A. (2011). Regulation of glycine receptor diffusion properties and gephyrin interactions by protein kinase C. EMBO J. 30, 3842–3853. 10.1038/emboj.2011.276 21829170 PMC3173796

[B88] Specht C. G. Izeddin I. Rodriguez P. C. El Beheiry M. Rostaing P. Darzacq X.. (2013). Quantitative nanoscopy of inhibitory synapses: counting gephyrin molecules and receptor binding sites. Neuron 79, 308–321. 10.1016/j.neuron.2013.05.013 23889935

[B89] Stepanenko O. V. Stepanenko O. V. Kuznetsova I. M. Verkhusha V. V. Turoverov K. K. (2013). Beta-barrel scaffold of fluorescent proteins: folding, stability and role in chromophore formation. Int. Rev. Cell Mol. Biol. 302, 221–278. 10.1016/B978-0-12-407699-0.00004-2 23351712 PMC3739439

[B90] Tanaka H. Hirano T. (2012). Visualization of subunit-specific delivery of glutamate receptors to postsynaptic membrane during hippocampal long-term potentiation. Cell Rep. 1, 291–298. 10.1016/j.celrep.2012.02.004 22832222

[B91] Tanudji M. Hevi S. Chuck S. L. (2002). Improperly folded green fluorescent protein is secreted *via* a non-classical pathway. J. Cell Sci. 115, 3849–3857. 10.1242/jcs.00047 12235295

[B92] Tardin C. Cognet L. Bats C. Lounis B. Choquet D. (2003). Direct imaging of lateral movements of AMPA receptors inside synapses. EMBO J. 22, 4656–4665. 10.1093/emboj/cdg463 12970178 PMC212729

[B93] Thomas P. Mortensen M. Hosie A. M. Smart T. G. (2005). Dynamic mobility of functional GABA_A_ receptors at inhibitory synapses. Nat. Neurosci. 8, 889–897. 10.1038/nn1483 15951809

[B94] Tretter V. Jacob T. C. Mukherjee J. Fritschy J.-M. Pangalos M. N. Moss S. J. (2008). The clustering of GABA_A_ receptor subtypes at inhibitory synapses is facilitated *via* the direct binding of receptor 2 subunits to gephyrin. J. Neurosci. 28, 1356–1365. 10.1523/JNEUROSCI.5050-07.2008 18256255 PMC6671568

[B95] Turrigiano G. G. (2000). AMPA receptors unbound: membrane cycling and synaptic plasticity. Neuron 26, 5–8. 10.1016/s0896-6273(00)81131-9 10798386

[B96] Yudowski G. A. Puthenveedu M. A. Leonoudakis D. Panicker S. Thorn K. S. Beattie E. C.. (2007). Real-time imaging of discrete exocytic events mediating surface delivery of AMPA receptors. J. Neurosci. 27, 11112–11121. 10.1523/JNEUROSCI.2465-07.2007 17928453 PMC3249441

